# Gender Parity Remains To Be Achieved for the Range of Editorial Roles Associated with Current Australasian Medical Journals

**DOI:** 10.7759/cureus.7879

**Published:** 2020-04-28

**Authors:** Deborah Verran, Karen Dwyer, Ruth Hardstaff, Paul Lawton, Helen Schultz

**Affiliations:** 1 Surgery, Ramsey Healthcare, Sydney, AUS; 2 Medicolegal Services, Avant Mutual Group, Sydney, AUS; 3 Medicine, Deakin University, Melbourne, AUS; 4 Surgery, John Hunter Hospital, Newcastle, AUS; 5 Nephrology, Menzies School of Health Research, Darwin, AUS; 6 Renal Services, Top End Health Services, Darwin, AUS; 7 Psychiatry, Private Practice, Melbourne, AUS

**Keywords:** gender, parity, medical, journals, editors, equity, data

## Abstract

With gender parity of medical school graduates having been achieved for well over two decades, it is timely to assess whether this has translated into gender parity for all of the editorial type roles of Australasian medical journals, reflecting a move toward gender equity. Data analysis was undertaken of the gender ratios of the current editorial roles of Australasian medical journals as compared to available Australian Health Workforce data. This reveals some variation in the gender ratios for all of the current range of editorial type positions and, hence, an absence of parity. There are no women holding formal editorial positions at all for 27.7% of these journals, whilst 77.7% of the chief editors’ roles are occupied by men. For five out of 18 (27.7%) of the journals, gender parity has been or is close to having been achieved for these particular roles. These gender ratios do not mirror the gender ratios of the wider community of practice for at least 50% of the journals. Hence, it can be seen that gender parity is yet to be achieved for the range of editorial roles of Australasian medical journals, which carries implications for gender equity initiatives.

## Introduction

Within Australia, the percentage of women graduating from medical school has been at least 50% for well over two decades, with the majority going on to enter clinical practice as fully qualified sub-specialized medical practitioners [1-2]. Despite these increasing numbers of women doctors entering the workforce, barriers to career progression particularly within some specialties continue to exist [3-4].

As recently identified, including by The Lancet Women initiative, gender equity is important for medicine, science, and global health and, ultimately, the community in general in order to achieve the best outcomes [5-6]. Hence, it is important to not only identify the various barriers to career progression that exist especially for women doctors but to also move formally to address how best to overcome them. One approach is to ensure that there is equity in opportunity for both genders in obtaining the traditional senior roles associated with medical leadership and/or academic activity [7]. This also pertains to the leadership roles that currently exist within professional organizations, along with other related professional activities [3,8]. One such professional activity is the undertaking of editorial roles for medical journals, where there have been ongoing concerns raised about the lack of women either in formal editorial roles or on the wider editorial boards [9-10]. Most recently, one prominent medical journal, The Lancet, has taken formal steps towards achieving gender equity in all of its related publishing activities [11].

We examined the composition of the editorial teams and boards of Australasian medical journals and compared them to health workforce data to see if gender parity has been achieved. This includes whether or not this has also translated into active policy development and implementation which also has implications for achieving gender equity.

## Materials and methods

An online search was undertaken of the gender composition of the formal editorial positions along with the associated editorial boards and/or related advisory groups of all the Australian and Australasian scientific journals with a focus on medicine and/or its subspecialties. Of note, medical journals published on behalf of institutes or fully open-access journals were excluded from this particular analysis. Gender was verified for all individuals by manually cross-checking with available online biographical information.

The primary outcome measures used were the absolute number and percentage of women versus men holding formal editorial positions. The following were considered a formal editorial position: A) Chief Editor, B) Any editorial position associated with a formal title. Secondary outcome measures were numbers and percentages of the editorial board or wider editorial advisory board members who are women versus men in general. A search was also undertaken of each journal’s public web site for any evidence of a public statement or policy pertaining to gender equity.

All of the relevant journal websites were accessed during a 30-day period from mid-August to mid-September 2019. A comparison was undertaken with the published data from the Australian Government Health Workforce (HW) dataset, where data were available for the gender ratios of fully qualified medical practitioners in the relevant specialty [2]. Doctors in training, along with medical students, were excluded from this analysis because it has traditionally been unusual for them to hold positions on editorial advisory boards.

## Results

A total of 18 journals were identified (see Appendix), with an editorial board consisting of members who reside and/or practice largely in Australia (and New Zealand where applicable). Of these, nine out of 18 (50%) had an Australasian focus, partly reflecting the current status of many of the subspecialty societies/organizations having members located across both Australia and New Zealand. Currently, six (33.3%) of the journals are published within Australia whilst 12 (66.6%) of the journals are published by known international medical publishers.

The most recent Australian HW data published of both the numbers of specialists in related subspecialties, along with general practitioners in Australia in 2017 according to gender, are summarized in Table 1. All practice groups have a number of fully qualified women who are potentially available as candidates for editorial roles. This does not take into consideration the ongoing flow-on effect of the increasing numbers of women undertaking specialty training where, by 2017, just over half of the specialty training positions in Australia were filled by women [2].

**Table 1:** Current proportion of fully qualified medical practitioners in Australia by clinical grouping according to gender (Health Workforce Data)

Clinical Group	Number	Female N (%)	Male N (%)
Anaesthesia	4430	1379 (31%)	3051 (69%)
Psychiatry	3498	1392 (39.8%)	2106 (60.2%)
Diagnostic radiology	2071	564 (27%)	1507 (73%)
Emergency medicine	1949	693 (35.5%)	1256 (64.5%)
Specialist O & G	1819	841 (46 %)	978 (55%)
General surgery	1680	269 (16%)	1411 (84%)
Paediatrics & child health	2078	1095 (52.6%)	983 (47.4%)
Cardiology	1234	174 (14.1%)	1060 (85.9%)
General medicine	997	226 (22.6%)	771 (77.4%)
Geriatric medicine	654	316 (48.3%)	338 (51.7%)
Endocrinology	604	322 (53.3%)	282 (46.7%)
Medical oncology	599	254 (42.4%)	345 (57.6%)
Public Health	257	122 (47.5%)	135 (52.5%)
Dermatology	508	229 (45%)	279 (55%)
Radiation oncology	363	157 (43.2%)	206 (56.8%)
General practice	24,088	10,098 (41.9%)	13,990 (58.1%)
Totals	46,829	18,131 (38.8%)	28,698 (61.2%)

The numbers of individuals holding either the chief or other types of what can be considered formal editors’ positions for each of these journals are depicted in Table 2 according to gender, with the relevant available HW data as a comparator. There is a wide range in the number of positions currently being occupied by women, with the mean being 33.9 %. Notably, 13/18 (72.2%) of these journals do not have any women in a Chief Editor position whilst 5/18 (27.7%) of the journals have no women in a formal editorial position. This does not reflect the gender ratio of the relevant community of practice in close to half (8/18; 45%) of these journals.

**Table 2:** Numbers of individuals holding Chief Editor and senior editorial roles according to gender compared to Health Workforce data

Journal Title	Female N = 37 (32.4%)	Male N = 77 (67.5%)	HW data Female (%)
Australian and New Zealand Journal of Obstetrics and Gynaecology	1 (100 %)	0 (0%)	46
Australian and New Zealand Journal of Public Health	4 (57%)	3 (43%)	47.5
Australian and New Zealand Journal of Surgery	0 (0%)	8 (100%)	16
Australian Rural and Remote Health	1 (100%)	0 (0%)	-
Australian and New Zealand Journal of Psychiatry	1 (12.5%)	7 (87.5%)	39.8
Australasian Journal of Ageing	6 (50%)	6 (50%)	48.3
Emergency Medicine Australia	0 (0%)	1 (100%)	35.5
Medical Journal of Australia	3 (50%)	3 (50%)	38.8
Australasian Journal of Dermatology	0 (0%)	2 (100%)	45
Health Promotion Journal of Australia	4 (80%)	1 (20%)	-
Australian Journal of Primary Health	7 (87.5%)	1 (12.5%)	41.9
Australasian Psychiatry	0 (0%)	3 (100%)	39.8
Journal of Medical Imaging and Radiation Oncology	0 (0%)	3 (100%)	27 43.2
Australian Journal General Practice	2 (50%)	2 (50%)	41.9
Australian Health Review	3 (60%)	2 (40%)	-
Internal Medicine Journal	0 (0%)	3 (100%)	21.4
Journal Paediatrics and Child Health	1 (16.6%)	5 (83.4%)	53
Heart Lung and Circulation	4 (13%)	27 (87%)	14

The composition of the wider editorial boards/advisory groups of these same journals are then depicted in Table 3 according to gender. There is a wide range seen in the number of these types of positions, varying from zero to 39 in number. The total number of positions currently occupied by women averages out at 33.5% (range 8.6 - 79%) with a slightly higher percentage of women on the editorial boards/advisory groups as compared to the other senior editorial positions. For five out of 18 (27.7%) of the journals, gender parity has been or is close to having been achieved for these particular roles. However, there is still some variation in these gender ratios that does not always reflect the percentage of women in practice when compared with the HW data. For five journals, the difference in the percentage of women holding such roles was less than a 5% difference as compared to the percentage of women in the wider workforce, whilst in another five journals, this percentage difference was less than 10%. However, for four out of 18 journals (22%), this percentage difference was more marked, whilst in four journals, a representative comparator from the HW data was not able to be ascertained.

**Table 3:** Numbers of individuals on editorial advisory boards according to gender compared to HW data

Journal Title	Female N = 134 (33.5%)	Male N = 266 (66.5%)	HW Data Female (%)
Australian and New Zealand Journal of Obstetrics And Gynaecology	8 (50%)	8 (50%)	46
Australian and New Zealand Journal of Public Health	7 (35%)	13 (65%)	47.5
Australian and New Zealand Journal of Surgery	4 (12.5%)	28 (87.5%)	16
Australian Rural and Remote Health	6 (46%)	7 (54%)	-
Australian and New Zealand Journal of Psychiatry	7 (21%)	26 (79%)	39.8
Australasian Journal of Ageing	7 (54%)	6 (46%)	48.3
Emergency Medicine Australia	14 (36%)	25 (64%)	35.5
Medical Journal of Australia	13 (35%)	24 (65%)	38.8
Australasian Journal of Dermatology	8 (38%)	13 (62%)	45
Health Promotion Journal of Australia	6 (75%)	2 (25%)	-
Australian Journal of Primary Health	11 (79%)	3 (21%)	41.9
Australasian Psychiatry	5 (36%)	9 (64%)	39.8
Journal of Medical Imaging and Radiation Oncology	12 (36%)	21(64%)	27 43.2
Australian Journal General Practice	Nil listed	Nil Listed	41.9
Australian Health Review	1 (25%)	3 (75%)	-
Internal Medicine Journal	8 (27%)	22 (73%)	21.4
Journal Paediatrics and Child Health	14 (37%)	24 (63%)	53
Heart Lung and Circulation	3 (8.6%)	32 (91.4%)	14

None of the journals had either a statement or a policy pertaining to gender parity, gender equity, or diversity situated either on their public website or accessible via a link. The majority of information that is available via the public websites is largely process focussed and technical in nature, directed predominantly at reviewers and manuscript authors. However, there was one recent editorial in the Australian and New Zealand Journal of Obstetrics and Gynaecology where the current status of gender equity within the specialty was explored in depth [12].

## Discussion

This analysis reveals that a number of the Australasian medical journals are close to achieving gender parity with respect to the composition of their editorial boards, but that as yet, this is not the case for all. Of greater concern is the lack of gender balance for either the Chief or other formal editorial positions. Currently, five journals have no women in these positions, which does not reflect the gender ratios for the respective wider professional community of interest at large in Australia as reflected in the HW data [2]. This is concerning because imbalances in editorial role gender representation contribute to disparities between the genders for both scientific publishing as well as career advancement activities [9,13].

As the HW comparator data are now two to three years old, this may not fully account for the increasing numbers of women qualifying and completing postgraduate training in recent years and may, in fact, underestimate the current gender ratios. In addition, some New Zealanders may hold editorial positions for the seven Australasian journals, which is not easily quantifiable. Plus, the limited amount of publicly accessible information may not reflect the current situation with respect to the gender ratios of these particular editorial positions. There is also a lack of publicly available summary information on the gender ratios for manuscript reviewers as well as for the authors of invited commentaries and editorials. These are other areas where, potentially, there may be a gender imbalance that may not reflect the current gender composition of the wider community of practice [14].

The lack of any public reference to either statements and/or policies pertaining to gender parity or equity on the journal websites may reflect any one of a number of issues. The majority of the current information on journal websites is technical in nature and geared towards relaying information to both reviewers and manuscript authors. There may be website constraints imposed by the publisher, the relevant statement may have been published elsewhere and, hence, is not readily accessible online via the journal, or there may be an acceptance of the ongoing status quo by the editorial team. This may reflect unconscious or implicit bias or may, instead, be a symptom of the constraints posed by the model of business practice including that many editorial roles involve pro bono work being undertaken [15-16]. It is increasingly being recognized that such statements are important and form part of a relatively easy, so-called ‘fix,’ with respect to medical journals achieving gender equity [17]. With one high-profile medical journal, The Lancet recently going down this path, it seems likely that others will now follow.

What is the significance of these findings? With women now making up just over half of the medical school graduates along with also occupying a significant percentage of the formal training positions in many specialties here in Australia, it is important to signal that they can also attain leadership roles [5,18]. With it now also being understood that academic knowledge production is influenced by gender and that a gender imbalance in editorial positions can unwittingly contribute to ongoing inequalities and prejudice, it is important that this issue be raised [19]. Hence, the importance of the data obtained from this analysis of this particular aspect of the current Australasian medical journal landscape. Permitting the status quo to continue could potentially now be viewed as unwittingly contributing to the barriers that women doctors may face in both sustaining an academic career and/or attaining leadership roles.

This finding carries other implications for medical journals on how best to now address the existing gender imbalances for these editorial type positions. This is because the first step towards ultimately achieving gender parity along with diversity for all of these editorial roles is by ensuring that there is a more appropriate representation of the women from the wider professional community of practice in each case [20]. This may involve setting targets for gender representation in each particular case until, eventually, parity is achieved, depending on the context, along with actively soliciting suitably qualified individuals to take up these positions [21]. In addition, serious thought needs to be given to collecting, tracking, and analyzing data according to gender for all activities pertaining to the publication processes for each particular journal and then publishing this data in the public domain [19,21-22]. This is in order to both establish and achieve transparency with respect to the journal manuscript-handling processes. The ultimate goal is for gender parity to be achieved across the board for all medical journal activities akin to what has been recently signaled by The Lancet, along with several other healthcare-related journals [11,21,23]. Several of The Lancet’s stable of journals are now beginning to publish data on what stage they have each reached with respect to achieving gender equity [24]. Ultimately, editorial board diversity should reflect the wider community at large [25].

## Conclusions

This retrospective analysis of a snapshot of the information publicly available on Australasian medical journal websites during the second half of 2019 has revealed that there is a lack of uniformity with respect to the gender ratios of men versus women holding editorial roles. In some cases, there are no women holding a formal editorial role at all, whilst gender parity is close to being achieved for the wider editorial roles in just under a third of cases. This has implications, including with respect to attaining gender equity, for this particular aspect of academic type endeavors.

## Appendices

List of journals whose websites were accessed

1. Australian and New Zealand Journal of Obstetrics and Gynaecology

2. Australian and New Zealand Journal of Public Health

3. Australian and New Zealand Journal of Surgery

4. Australian Rural and Remote Health

5. Australian and New Zealand Journal of Psychiatry

6. Australasian Journal of Ageing

7. Emergency Medicine Australia

8. Medical Journal of Australia

9. Australasian Journal of Dermatology

10. Health Promotion Journal of Australia

11. Australian Journal of Primary Health

12. Australasian Psychiatry

13. Journal of Medical Imaging and Radiation Oncology

14. Australian Journal of General Practice (Previously Australian Family Physician)

15. Australian Health Review

16. Internal Medicine Journal (RACP)

17. The Journal of Paediatrics and Child Health

18. Heart Lung and Circulation
